# Where the “*ruber*” Meets the Road: Using the Genome of the Red Diamond Rattlesnake to Unravel the Evolutionary Processes Driving Venom Evolution

**DOI:** 10.1093/gbe/evae198

**Published:** 2024-09-10

**Authors:** Samuel R Hirst, Rhett M Rautsaw, Cameron M VanHorn, Marc A Beer, Preston J McDonald, Ramsés Alejandro Rosales García, Bruno Rodriguez Lopez, Alexandra Rubio Rincón, Hector Franz Chávez, Víctor Vásquez-Cruz, Alfonso Kelly Hernández, Andrew Storfer, Miguel Borja, Gamaliel Castañeda-Gaytán, Paul B Frandsen, Christopher L Parkinson, Jason L Strickland, Mark J Margres

**Affiliations:** Department of Integrative Biology, University of South Florida, Tampa, FL, USA; Department of Integrative Biology, University of South Florida, Tampa, FL, USA; School of Biological Sciences, Washington State University, Pullman, WA, USA; Department of Integrative Biology, University of South Florida, Tampa, FL, USA; School of Biological Sciences, Washington State University, Pullman, WA, USA; Department of Integrative Biology, University of South Florida, Tampa, FL, USA; Biological Sciences Department, Clemson University, Clemson, SC, USA; Facultad de Ciencias Biológicas, Universidad Juárez del Estado de Durango, Durango, Mexico; Facultad de Ciencias Biológicas, Universidad Juárez del Estado de Durango, Durango, Mexico; Herp.MX©, Colima, Mexico; Facultad de Ciencias Biológicas y Agropecuarias, Universidad Veracruzana, Veracruz, Mexico; PIMVS Herpetario Palancoatl, Veracruz, Mexico; PIMVS Herpetario Palancoatl, Veracruz, Mexico; School of Biological Sciences, Washington State University, Pullman, WA, USA; Facultad de Ciencias Biológicas, Universidad Juárez del Estado de Durango, Durango, Mexico; Facultad de Ciencias Biológicas, Universidad Juárez del Estado de Durango, Durango, Mexico; Department of Plant and Wildlife Sciences, Brigham Young University, Provo, UT, USA; Biological Sciences Department, Clemson University, Clemson, SC, USA; Department of Biology, University of South Alabama, Mobile, AL, USA; Department of Integrative Biology, University of South Florida, Tampa, FL, USA

**Keywords:** transcriptomics, population genomics, ontogeny, venom

## Abstract

Understanding the proximate and ultimate causes of phenotypic variation is fundamental in evolutionary research, as such variation provides the substrate for selection to act upon. Although trait variation can arise due to selection, the importance of neutral processes is sometimes understudied. We presented the first reference-quality genome of the Red Diamond Rattlesnake (*Crotalus ruber*) and used range-wide ‘omic data to estimate the degree to which neutral and adaptive evolutionary processes shaped venom evolution. We characterized population structure and found substantial genetic differentiation across two populations, each with distinct demographic histories. We identified significant differentiation in venom expression across age classes with substantially reduced but discernible differentiation across populations. We then used conditional redundancy analysis to test whether venom expression variation was best predicted by neutral divergence patterns or geographically variable (a)biotic factors. Snake size was the most significant predictor of venom variation, with environment, prey availability, and neutral sequence variation also identified as significant factors, though to a lesser degree. By directly including neutrality in the model, our results confidently highlight the predominant, yet not singular, role of life history in shaping venom evolution.

**Significance**

Although the neutral theory of molecular evolution has provided a null model for >50 years when examining the genetics underlying phenotypes, neutral processes are not always explicitly incorporated into trait-based analyses. Snake venoms evolve quite rapidly and are often assumed to be evolving solely under strong directional selection. Here, we present the first reference-quality genome of the Red Diamond Rattlesnake and use range-wide ‘omic data to estimate the degree to which neutral and adaptive evolutionary processes shape venom evolution. We found that life-history evolution was the dominant force underlying venom variation. Following life history, however, neutral sequence variation explained comparable variation to both biotic and abiotic factors, suggesting that neutral processes play a more prominent role than previously thought.

## Introduction

Natural populations often exhibit exceptional degrees of phenotypic variation (Darwin 1859; Nevo 1978), such as body color of strawberry poison frogs (Summers et al. 2003; Yang et al. 2019), body and beak size of Galápagos Island finches (Darwin 1859; Grant and Grant 2002), and levels of salinity resistance in salt marsh plants (Hester et al. 2001) among others (Hoekstra et al. 2006; Dickson et al. 2017). Such variation can be the product of adaptive and/or neutral evolutionary processes (Lande 1976). Neutrality often serves as the evolutionary null hypothesis (Fisher 1930; Kimura 1968; Ohta 1973; Nei 2005; Müller et al. 2022), as it provides a baseline against which the effects of natural selection can be measured (Serra et al. 2013; Rohlfs et al. 2014; Zhang 2018). Phenotypic variation, however, is frequently explored solely within the framework of selection and adaptation (Gould and Lewontin 1979; Brodie et al. 2002; Williams et al. 2003; Hanifin et al. 2008; Smith et al. 2023), even when such variation may be the product of neutral evolutionary processes via geographically limited dispersal and consequent gene flow (Lande 1976; Alexander et al. 2006). Indeed, a textbook example of phenotypic variation assumed to be adaptive is toxin production in rough-skinned newts (*Taricha granulosa*). Newt toxin production may be a response to coevolutionary interactions with a toxin-resistant predator, the common garter snake (*Thamnophis sirtalis*; Brodie and Brodie 1990; Brodie et al. 2002, 2005; Williams et al. 2003, 2010). Recently, a robust statistical framework accounting for demographic histories and population structure demonstrated that *T. granulosa* toxicity levels were more significantly predicted by population structure and isolation-by-distance (IBD) rather than resistance levels of *T. sirtalis* (Hague et al. 2020), indicating that neutral evolutionary processes were substantially contributing to variation in toxin production. The relationship between population structure and toxin production in *T. granulosa* highlights the importance of determining whether other traits assumed to be evolving under strong selection actually exhibit patterns consistent with *only* adaptive evolution (Zhang 2018).

Recently, snake venom has emerged as an effective system for studying adaptive evolution (Margres et al. 2017a; Arbuckle 2020; Mason et al. 2022; Rao et al. 2022). However, neutral evolution in this system is occasionally untested (Sanz et al. 2006; Barlow et al. 2009; Cipriani et al. 2017; Smiley-Walters et al. 2017; Davies and Arbuckle 2019; Smith et al. 2023) despite evidence that neutral processes, such as genetic drift, may play a role in shaping venom characteristics (Sasa 1999; Aird et al. 2017; Casewell et al. 2020; Rao et al. 2022). Snake venom is a complex, polygenic trait composed of 40–100 proteinaceous toxins used for prey immobilization, digestion, and defense (Daltry et al. 1996; Barlow et al. 2009; Casewell et al. 2011; Mackessy 2021). Despite the complex genomic architecture of venom (Schield et al. 2019; Margres et al. 2021a; Hogan et al. 2024), toxin gene expression is specific to venom glands (Rokyta et al. 2015), with differences in expression having clear, functional effects on the venom phenotype (Barlow et al. 2009; Holding et al. 2016; Margres et al. 2017a; Smiley-Walters et al. 2017; Casewell et al. 2020). Venom expression exhibits extensive variation across different species (Casewell et al. 2014; Margres et al. 2015a; Jackson and Fry 2016; Jackson et al. 2016; Durban et al. 2017; Pla et al. 2019; Senji Laxme et al. 2019; Holding et al. 2021), populations of the same species (Massey et al. 2012; Margres et al. 2015a, 2019; Holding et al. 2018; Smith et al. 2023), and life histories (Andrade and Abe 1999; Alape-Girón et al. 2008; Barlow et al. 2009; Margres et al. 2015a, 2015b; Wray et al. 2015; Modahl et al. 2016; Cipriani et al. 2017; Durban et al. 2017; Rokyta et al. 2017; Borja et al. 2018; Schonour et al. 2020); venom expression variation at all three scales has also been shown to be the result of genetic rather than environmental (i.e. plastic) effects (Gibbs et al. 2009; Margres et al. 2015b). Abiotic and/or biotic selective pressures, such as differences in environment (Strickland et al. 2018; Margres et al. 2021b; Siqueira-Silva et al. 2021), diet (Mackessy et al. 2003; Holding et al. 2018; Schonour et al. 2020; Holding et al. 2021), or prey venom resistance (Barlow et al. 2009; Holding et al. 2016; Margres et al. 2017a), may produce such variation. Antagonistic coevolutionary interactions with prey have been associated with venom expression variation in certain cases (Barlow et al. 2009; Holding et al. 2016; Margres et al. 2017a); however, prey-driven selection is often assumed to produce venom expression variation without sufficient empirical evidence (e.g. Smith et al. 2023). Determining whether venom expression variation is adaptive requires both precise knowledge of diet and quantitative and functional measurements of venom effectiveness in multiple prey species and populations, making it exceptionally difficult to test (Barlow et al. 2009; Holding et al. 2016; Margres et al. 2017a; Smiley-Walters et al. 2017; Casewell et al. 2020). Consequently, venom studies often rely on methods for detecting signatures of selection such as *dN/dS* ratios (Juárez et al. 2008; Margres et al. 2013; Rokyta et al. 2013; Mason et al. 2020; Zhao et al. 2021), but changes to gene-expression patterns have, in general, been found to explain a disproportionate amount of venom expression variation (Margres et al. 2016a, 2017a, 2017b), consistent with other traits (Gompel et al. 2005; Fraser 2013; Konczal et al. 2015). Nevertheless, venom expression variation should not be exclusively attributed to adaptive evolution without investigating the potential role of neutral evolutionary processes (Sasa 1999; Casewell et al. 2020; Rao et al. 2022). Much like the variable toxin production observed across *T. granulosa* populations, geographic variation in snake venom expression may be erroneously attributed solely to selection, whereas it may arise, at least in part, from neutral evolutionary processes.

**Fig. 1.**
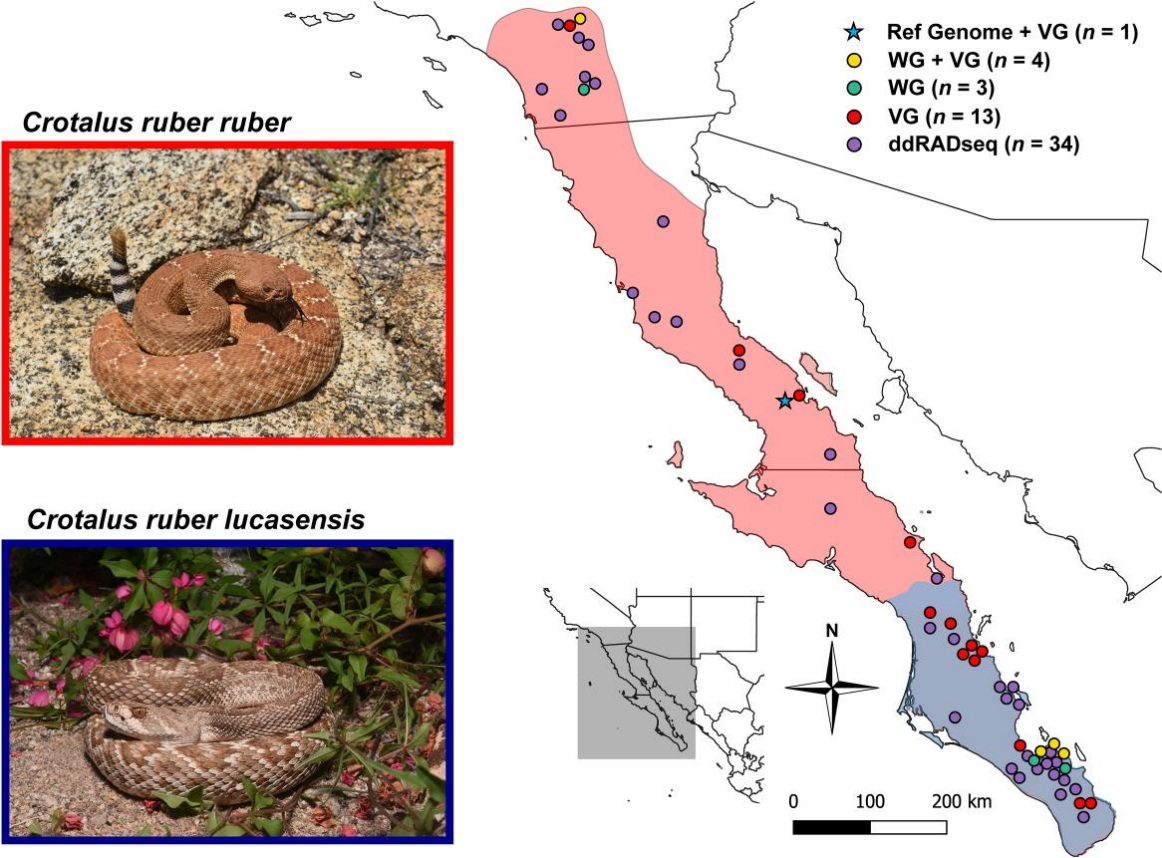
Distribution and sampling map of *Crotalus r. ruber* (red map shading) and *C. r. lucasensis* (blue map shading). Color of sampling point is based on the types of data generated for the individual sampled at that location. Ref Genome, PacBio HiFi genome sequencing; WG, short-read whole-genome sequencing; VG, venom-gland transcriptomes; ddRADseq, double digest restriction-site associated DNA Sequencing. Snake image credits: Ricardo Ramírez Chaparro.

The Red Diamond Rattlesnake (*Crotalus ruber*) exhibits ontogenetic and geographic venom variation (Straight et al. 1992), making it an excellent focal species for investigating the contributions of neutral and adaptive processes on snake venom evolution. *Crotalus ruber* is a large-bodied pitviper found in western North America ranging from San Bernadino County, California, USA, south throughout the Baja California peninsula and various islands. Habitat throughout its range varies extensively (Grismer 2002), and its prey composition, which includes primarily small- to medium-sized mammals, is well characterized (Dugan and Hayes 2012). Two mainland subspecies are recognized: *C. r. ruber* extends from the northern range edge to the central region of the Baja peninsula, and *C. r. lucasensis* inhabits the southern third of the Baja peninsula (Fig. 1). The current subspecies definitions are based on morphological (Grismer 2002) and genetic differentiation, with divergence occurring ∼570 ka before present (Harrington et al. 2018). Although *C. ruber* exhibits venom variation in specific protein families across its geographic range and life history (Straight et al. 1992; Pozas-Ocampo et al. 2020), variation across the complete venom phenotype as well as the evolutionary processes producing such variation have yet to be investigated.

In this study, we investigated the evolutionary processes, both adaptive and nonadaptive, that may have produced variation in a trait that is often assumed to be evolving under strong directional selection. We aimed to 1) generate the first reference *C. ruber* genome for use in downstream analyses, 2) characterize neutral population structure and demographic history, 3) quantify venom expression variation across populations and life-history stages, and 4) determine the relative contributions of neutral evolutionary processes, geographically variable abiotic and/or biotic factors, and life history in explaining venom expression evolution through robust statistical models. If venom is rapidly evolving due to selection, we expect decoupling of patterns produced by neutral evolutionary processes, such as population structure and IBD (Wright 1943; Williams et al. 1988; Keller et al. 2009), with venom variation spatially, as demonstrated previously (Margres et al. 2019). Specifically, we would expect patterns of venom variation to correlate with patterns of variation in abiotic and/or biotic factors such as dietary composition or climate (Holding et al. 2018). Conversely, if venom is evolving due to neutral processes, we expect a strong correlation between neutral sequence variation and venom variation, similar to what was found for toxin-production levels in newts (Hague et al. 2020). Overall, our approach integrating diverse data types from multiple individuals across the range will allow us to identify the most significant factors driving venom evolution within a species.

## Materials and Methods

### Sampling

We collected 21 *C. ruber* across the Baja California Peninsula, MX and southern California, USA (Fig. 1). Snakes were captured via road cruising or visual encounter surveys. Upon capture, sampling locality, snout–vent–length (SVL), tail length, and sex were recorded. Venom and blood were sampled in the field from two individuals prior to release. Nineteen individuals were euthanized, dissected, vouchered, and deposited at La Colección Herpetologica de la Facultad de Ciencias Biologicas de la Universidad Juárez del Estado de Durango in Gómez Palacio, Durango, MX. For dissection, we removed the right and left venom glands, heart, liver, gonad, kidney, muscle, and/or blood and stored each tissue in RNALater and/or 95% ethanol. Snakes were collected under the following permits: Secretaría de Medio Ambiente y Recursos Naturales Oficio N SGPA/DGVS/01090/17; SGPA/DGVS/002288/18; SGPA/DGVS/13338/19; SGPA/DGVS/2190/19; SGPA/DGVS/08831/20; SGPA/DGVS/10362/21 and California Department of Fish and Wildlife SC-12985. The procedures outlined were approved by the University of South Florida Institutional Animal Care and Use Committee (IACUC) under protocol IS00011949 and Clemson University IACUC protocol 2017-067.

### Reference Genome Sequencing and Assembly

A high-quality reference genome for *C. ruber* was produced from a subadult male (66.5 cm SVL, 71.0 cm TL) sampled near Bahía de los Ángeles, Baja California, MX (Fig. 1). High-molecular-weight (HMW) genomic DNA (gDNA) was obtained from blood extracted from the caudal vein. The genome was sequenced using Pacific Biosciences HiFi sequencing on 1.5 cells on the Sequel II sequencer at the University of Delaware Sequencing & Genotyping Center. We used HiFiAdapterFilt (Sim et al. 2022) to detect adapter contamination in the sequenced reads and found 1,259 reads (0.00094% of total) with adapters. We assembled the genome using all reads with the Hifiasm assembler (Cheng et al. 2021). We then used Blast (Johnson et al. 2008) with the UniVec database to detect adapters within the assembly and masked all adapter contaminants using the BEDTools maskfasta function (Dale et al. 2011). Assembly quality statistics were calculated using MERQURY (Rhie et al. 2020) and Genome Tools (Gremme et al. 2013). Assembly completeness was assessed using BUSCO (Simão et al. 2015) for datasets Vertebrata and Sauropsida. We screened for foreign contamination of the assembled genome using NCBI FCS-GX (Astashyn et al. 2024; Bush et al. 2024; Pozo et al. 2024). No contamination was detected in the genome assembly and classification of all contigs was consistent with the expected taxonomic composition of the target organism. To achieve a chromosomal representation of the assembly, we aligned the *C. ruber* genome to the *Crotalus adamanteus* genome (Hogan et al. 2024) using Ragtag (Alonge et al. 2022). A Circos plot of the genome was generated using the Circlize package (Gu et al. 2014) in R. Genome assembly and all data generated in this study are available at NCBI PRJNA1051499.

### Reference Genome Annotation

To aid in genome annotation, we generated transcriptomes for blood, gonad, heart, kidney, liver, and right and left venom glands from the same subadult male used for reference genome assembly (see below for details on RNA extraction and sequencing); all RNA-seq data were aligned to the genome using Hisat2 (Kim et al. 2019). The genome was then annotated using GeMoMa (Keilwagen et al. 2019) with the *Crotalus adamanteus* (Hogan et al. 2024) genome and the aligned *C. ruber* transcriptome data as references. Functional annotations were added using InterProScan (Jones et al. 2014) and Blast (Johnson et al. 2008). Due to the complex architecture of venom genes in large-tandem arrays, automated annotation of venom genes is often unreliable. As such, we used Geneious Prime (Kearse et al. 2012) and FGENESH+ (Salamov and Solovyev 2000) to manually identify and annotate venom genes as previously described (Margres et al. 2021a).

### ddRADseq Data Processing

We downloaded double digest restriction-site associated DNA (ddRADseq) data for 34 *C. ruber* from NCBI SRA (Fig. 1; PRJNA413434; Harrington et al. 2018). Nonreference based population genomic analyses can be prone to errors arising from repetitive regions, polymorphisms, and sequencing errors (Brandies et al. 2019); therefore, we reanalyzed the *C. ruber* ddRADseq data using reference-based alignment to the generated reference genome described above. All ddRADseq data were aligned to the reference genome using iP*γ*RAD (Eaton and Overcast 2020) using default parameters.

### Whole-Genome Sequencing Data Generation and Processing

We generated short-read whole-genome sequencing (WGS) data for six *C. ruber* (PRJNA1051499) and downloaded an additional *C. ruber* whole-genome from NCBI SRA (PRJNA593834; Schield et al. 2022). For the six genomes generated in this study, DNA was isolated from blood samples using the EZNA Tissue DNA Kit (Omega Bio-tek), and DNA libraries were generated using the Ultra II FS DNA Library Prep kit (New England Biolabs). Libraries were sequenced at the North Carolina State University Genomic Sciences Laboratory using Illumina Novaseq 6,000 with 150 paired-end sequencing (supplementary table S6, Supplementary Material online). Data were mapped to the reference genome using bowtie2 (Langmead and Salzberg 2012), and SNPs were called using GATK (McKenna et al. 2010) best practices workflow for germline short variant discovery with default parameters and recommended hard filters. A merged VCF file with the 34 ddRADseq samples and seven WGS samples was produced using bcftools merge and was subsequently filtered using VCFtools (Danecek et al. 2011) with the following parameters: minimum allele frequency (maf) 0.05, minimum depth (minDP) 5, and max-missing 0.5. The final combined genomic dataset included 41 individuals and 5,284 SNPs.

### Transcriptome Sequencing

We sequenced venom-gland transcriptomes from 12 individuals and additional blood, gonad, heart, kidney, and liver transcriptomes for the reference genome animal (PRJNA1051499) as outlined above. We also downloaded six additional venom-gland transcriptomes from NCBI SRA (PRJNA88989; Holding et al. 2021). Venom glands were processed following the approach of Rokyta et al. (2012). Briefly, for venom glands, venom was extracted four days prior to euthanasia to allow maximum transcription upon venom gland extraction (Rotenberg et al. 1971). At four days, snakes were euthanized and dissected. For dissection, the left and right venom glands, heart, blood, muscle, kidney, liver, and gonad were removed and placed in RNALater. We extracted RNA from the left and right venom glands separately, then combined in equal quantities for RNA library prep for each snake. For the reference genome snake, we also extracted RNA from each of the tissues listed above. We isolated RNA using a TRIzol extraction method as outlined in Rokyta et al. (2017). RNA libraries were generated using the Ultra II RNA Library Prep Kit for Illumina (New England Biolabs) and sequenced at the Florida State University DNA Sequencing Facility using NovaSeq 6,000 and the Oklahoma Medical Research Foundation Clinical Genomics Center using the NovaSeq X Plus with 150 paired-end sequencing (supplementary table S6, Supplementary Material online). Because gene expression values are sensitive to the read count methods employed, particularly for genes with exceptionally low and high expression (Liu et al. 2022), we mapped each transcriptome to the generated reference genome using Hisat2 (Kim et al. 2019) and estimated read counts for genes using both HTSeq-count (Anders et al. 2015; Putri et al. 2022) and Stringtie2 (Pertea et al. 2015). We used these two read-count estimation methods to provide complementary yet distinct quantitative estimates of gene expression to account for potential biases inherent in each approach. StringTie2 assembles RNA transcripts and estimates gene expression based on these assembled transcripts. HTSeq-counts directly counts the number of reads mapped to predefined features (e.g. genes labeled in a GFF3 annotation file), providing a direct measure of gene expression but potentially overlooking transcript complexity, such as alternative splicing or multiple isoforms, which may be better accounted for by StringTie2.

### Estimating Population Structure and Neutral Genetic Divergence

To recharacterize *C. ruber* population structure (Harrington et al. 2018), we used conStruct (Bradburd et al. 2018) on the combined genomic dataset (n=41) described above. We removed SNPs with >30% missing data and subsequently removed two individuals with >50% missing data for a reduced dataset containing 39 individuals and 2,241 SNPs. We initially tested K=1--5 genetic clusters using both spatial and nonspatial models and compared predictive accuracies using cross-validation. For each value of *K* and each type of model, we ran cross-validation using 20 replicates and 10,000 iterations, with SNPs split into 75% training and 25% testing data partitions. We ran each model for 20,000 iterations using three independent MCMC replicates. Additionally, we investigated patterns of sequence dissimilarity across all individuals and SNPS (n=41; 5,284 SNPs) using principal coordinate analysis (PCoA) from the R package dartR (Gruber et al. 2018). We then calculated ${\text{F}}_{ST}$ between the defined populations using VCFtools (Danecek et al. 2011) on both the full (n=41; 5,284 SNPs) and reduced (n=39; 2,241 SNPs) genomic dataset.

### Estimating Effective Migration Surfaces

To infer migration rates in *C. ruber*, we used EEMS (Petkova et al. 2016) on the full combined genomic dataset (n=41; 5,284 SNPs). We converted the merged WGS and ddRADseq SNP dataset to PLINK format (Purcell et al. 2007) and transformed the data to a pairwise distance matrix using “bed2diffs” function in EEMS. We used EEMS to estimate migration surfaces by running three independent chains, each with 1,000 demes, 10,000,000 MCMC iterations, 1,000,000 iterations of burn-in, and a thinning interval of 10,000. All chains successfully converged (supplementary fig. S5, Supplementary Material online).

### Estimating Demographic History

To estimate effective population size ($N_{e}$) through time for each *C. ruber* population as identified in conStruct above, we used pairwise sequentially Markovian coalescence (PSMC; Li and Durbin 2011). We used PSMC over similar methods (e.g. MSMC, SMC++, Stairway Plot; Schiffels and Durbin 2014; Liu and Fu 2015; Terhorst et al. 2017) due to its higher precision and accuracy, especially during intermediate (∼10,000–666 generations) time periods (Patton et al. 2019); however, PSMC may imprecisely estimate $\left(N_{e}\right)$ towards the present (Liu and Fu 2015; Nadachowska-Brzyska et al. 2016; Patton et al. 2019). Therefore, interpretations of historical demographic history based on our analyses were limited to intermediate evolutionary timescales as defined above. We inferred $N_{e}$ across 28 free atomic time intervals (4+25*2+4+6) and checked for variance in $N_{e}$ estimation by performing 100 bootstrap replicates (supplementary fig. S6, Supplementary Material online). We used the published generation time (g=3.3) and mutation rate ($\mu =0.7\;x\;10^{-8}$) of sister taxon *Crotalus atrox* (Castoe et al. 2007; Holding et al. 2021).

### Venom Proteomics

To characterize *C. ruber* venom variation, we collected venom from 20 individuals and used reversed-phase high performance liquid chromatography (RP-HPLC) to quantify venom protein expression. Venom was collected and then dried and stored at −80^∘^C prior to analysis. We conducted RP-HPLC on a Dionex ultimate 3000 UHPLC DAD (Thermo Fisher Scientific) and a BeckmanSystem Gold HPLC (BeckmanCoulter) using a Jupiter 5 *μ*m C18 300 Å, LC Column 250 *times* 2 mm, Ea column. 50 *μ*g of total venom protein were injected onto the column using a solvent system of A=0.1% trifluoroacetic acid (TFA) in water and B = 0.075% TFA in acetonitrile. After five minutes at 5% B, a 1% per minute linear gradient of A and B was run to 25% B, followed by a 0.25% per minute gradient from 25% to 65% B at a flow rate of 0.6 mL per min (Margres et al. 2014). Column effluent was monitored at 220 nm. RP-HPLC peaks were quantified in the Chromeleon software (Thermo Fisher Scientific). To estimate the relative abundance of each protein peak, we measured the area under the peak relative to the total area of all peaks identified (Gibbs and Rossiter 2008). Prior to statistical analyses, quantified peaks were transformed in R using isometric Log-Ratio (ILR) from the rombCompositions package (Templ et al. 2023).

### Characterizing Venom Expression Differentiation

To identify patterns of venom expression variation, we first conducted a PCA on the ILR transformed venom proteomic data (n=20) in R using the “prcomp” function from the Stats package. We then conducted a simple regression model (“lm” function in R) comparing PC1 with SVL to test for the effects of ontogeny, which is common in rattlesnakes (Andrade and Abe 1999; Alape-Girón et al. 2008; Barlow et al. 2009; Margres et al. 2015a, 2015b; Wray et al. 2015; Modahl et al. 2016; Cipriani et al. 2017; Durban et al. 2017; Rokyta et al. 2017; Borja et al. 2018; Schonour et al. 2020). To determine whether venom protein expression was significantly different across populations and/or age classes, we performed a permutational multivariate analysis of variance (PERMANOVA) in the “adonis2” function of the vegan package (Oksanen et al. 2020) on the ILR transformed venom proteomic data. The same approach using PCA, simple regression, and PERMANOVA was repeated using normalized venom-gland transcriptomic data from HTSeq-count (n=18; Anders et al. 2015; Putri et al. 2022) to verify concordance between venom proteomic and venom-gland transcriptomic data. Read count data from HTSeq-count were normalized using median of ratios from DEseq2 (Anders and Huber 2010).

We also tested whether specific toxin transcripts were significantly differentially expressed (DE) across populations and/or age classes using the program DESeq2 (Love et al. 2014) on our venom-gland transcriptome data (n=18). For the geographic comparison, we used the two populations as delineated from conStruct (Bradburd et al. 2018) and accounted for ontogeny in the model by using age class as a covariate. For the ontogenetic comparison, we accounted for geography in the model by including population as a covariate. Significance in differential expression was calculated using the FDR-adjusted *P* value (*P*adj) and log2 fold change (LFC) ≥ 1 from DESeq2.

### Determining the Contributions of Ecological and Evolutionary Factors on Venom Expression Variation through Conditional Redundancy Analysis

To estimate the contributions of neutral processes, life history (i.e. snake size), prey availability and diversity, and climactic conditions on *C. ruber* venom expression variation, we used conditional Redundancy Analyses (RDA; van den Wollenberg 1977; Liu 1997; Capblancq and Forester 2021). Briefly, conditional RDA controls for the effects of one set of explanatory variables prior to conducting RDA on the residual matrix. RDA functions as an extension to multiple regression analysis but permits multivariate response variables. Significance testing within an RDA framework utilizes permutation, making it robust to small sample size and distributional assumptions (Liu 1997).

Here, we explored venom expression variation using eight different response variables: (i) estimated read counts for all toxin genes using HTSeq-counts (Anders et al. 2015; Putri et al. 2022), (ii) estimated read counts for all toxin genes using Stringtie2 (Pertea et al. 2015) and (iii–viii) estimated read counts for specific paralogs belonging to the six dominant toxin families individually using HTSeq-counts. All venom response variables were multivariate toxin gene expression data representing the abundance levels of multiple toxin loci, enabling us to identify the most significant explanatory variables influencing the expression of toxin genes within a multivariate framework. Prior to analyses, we transformed read count data using the median of ratios in DESeq2 (Anders and Huber 2010). We conditioned each explanatory variable (nontoxin sequence variation, toxin sequence variation, climactic variation, prey availability, and prey diversity, each described below) in the model on the other explanatory variables to remove the potential confounding effects for each. We then conducted a marginal test using all explanatory variables and used forward model selection to generate the marginal model (i.e. best model). Conditional RDAs were conducted using the “rda” function from the Vegan package in R (Oksanen et al. 2020) and included the “anova” function for significance testing, “RsquareAdj” for model fit, and “ordiR2step” for forward model selection. We describe each explanatory variable below:

1. To include the contributions of neutral processes in the model, we generated a SNP dataset for nontoxin genes, our proxy for neutrality (Rautsaw et al. 2019; Holding et al. 2021), sequenced from the venom-gland transcriptomes (n=18). We used GATK (McKenna et al. 2010) with default parameters as previously outlined. Additional filtering parameters from VCFtools (Danecek et al. 2011) included min-alleles 2, minDP 5, max-missing 0.5, and minimum allele frequency of 0.1. We converted our annotated reference genome file to a BED file and used VCFTools with functions “bed” and “exclude-bed” to isolate nontoxin genes from toxin genes, resulting in 41,236 nontoxin SNPS for analysis. We also attempted to remove potential signatures of selection from the nontoxin SNP data by creating a second dataset containing only synonymous sites. Variant annotation was conducted using SnpEff (Cingolani et al. 2012), resulting in 3,818 nontoxin synonymous SNPs. Nontoxin sequence variation was summarized using principal Coordinate Analysis (PCoA) from the R package dartR (Gruber et al. 2018) on both the full nontoxin SNP dataset (41,236 SNPs) and the nontoxin synonymous SNP dataset (3,818 SNPs; supplementary fig. S2B-C, Supplementary Material online). To determine whether the inclusion of other nontoxin SNP types (nonsynonmous and intronic) accurately represented neutral genetic divergence, we conducted a regression using PCo1 of the full nontoxin SNP dataset and PCo1 of the nontoxin synonymous SNP dataset (supplementary fig. S4, Supplementary Material online). We retained PCo1 and PCo2 of the full nontoxin SNP dataset (41,236 SNPs) for use in the conditional RDAs (supplementary table S7, Supplementary Material online).
2. To include signatures of selection on toxin gene sequences, we summarized toxin sequence variation from venom-gland transcriptomes (n=18) following the same approach above; however, following filtration, toxin genes were isolated from nontoxin genes, resulting in a toxin-only SNP dataset of 1,760 SNPs. Note that toxin sequence variation was excluded as a variable in individual toxin families due to the limited number of independent SNPs for each family (supplementary fig. S2D and table S7, Supplementary Material online).
3. Abiotic factors were incorporated using differing environmental conditions as represented by the 19 Worldclim Bioclim variables (Hijmans et al. 2005) at each sampling site using 5 min spatial resolution. We conducted a PCA across the data, and PC1 and PC2 were retained for use in the conditional RDAs (See supplementary table S4, Supplementary Material online for PC loadings and proportion of variance explained by each PC).
4. To account for potential differences in diet between individuals, we incorporated prey availability in the model following the approach of Holding et al. (2018). Prey availability was determined using published accounts of prey data for *C. ruber* (Klauber 1997; Clark et al. 2012; Dugan and Hayes 2012; Holding et al. 2021) resulting in 29 known prey items (supplementary table S5, Supplementary Material online). Geographic range was determined for each prey item using iNaturalist (www.inaturalist.org), IUCN (www.iucn.org), and/or Map of Life (mol.org). For each sample site, each prey item was given a value of “1” if present and “0” if absent (supplementary table S7, Supplementary Material online). We conducted Nonmetric multidimensional scaling (NMDS) on the prey dataset using the “metaMDS” function from the Vegan package in R (Oksanen et al. 2020) and retained MDS1 and MDS2 for use in the conditional RDAs (See supplementary table S5, Supplementary Material online for NMDS loadings and proportion of variance explained by each MDS).
5. Phylogenetic diversity of prey has been shown to predict patterns of venom evolution across species (Holding et al. 2021); therefore, we incorporated estimates of prey mean phylogenetic distance (MPD) in the model. We generated a phylogeny of the 29 *C. ruber* prey items using www.timetree.org (supplementary fig. S7, Supplementary Material online; Kumar et al. 2017) and used the “ses.mpd” function from the Picante R package (Kembel et al. 2010) to calculate MPD at each site (supplementary table S8, Supplementary Material online).

See supplementary table S7, Supplementary Material online for data used in conditional RDAs.

## Results

### De Novo Genome Assembly and Annotation

**Fig. 2.**
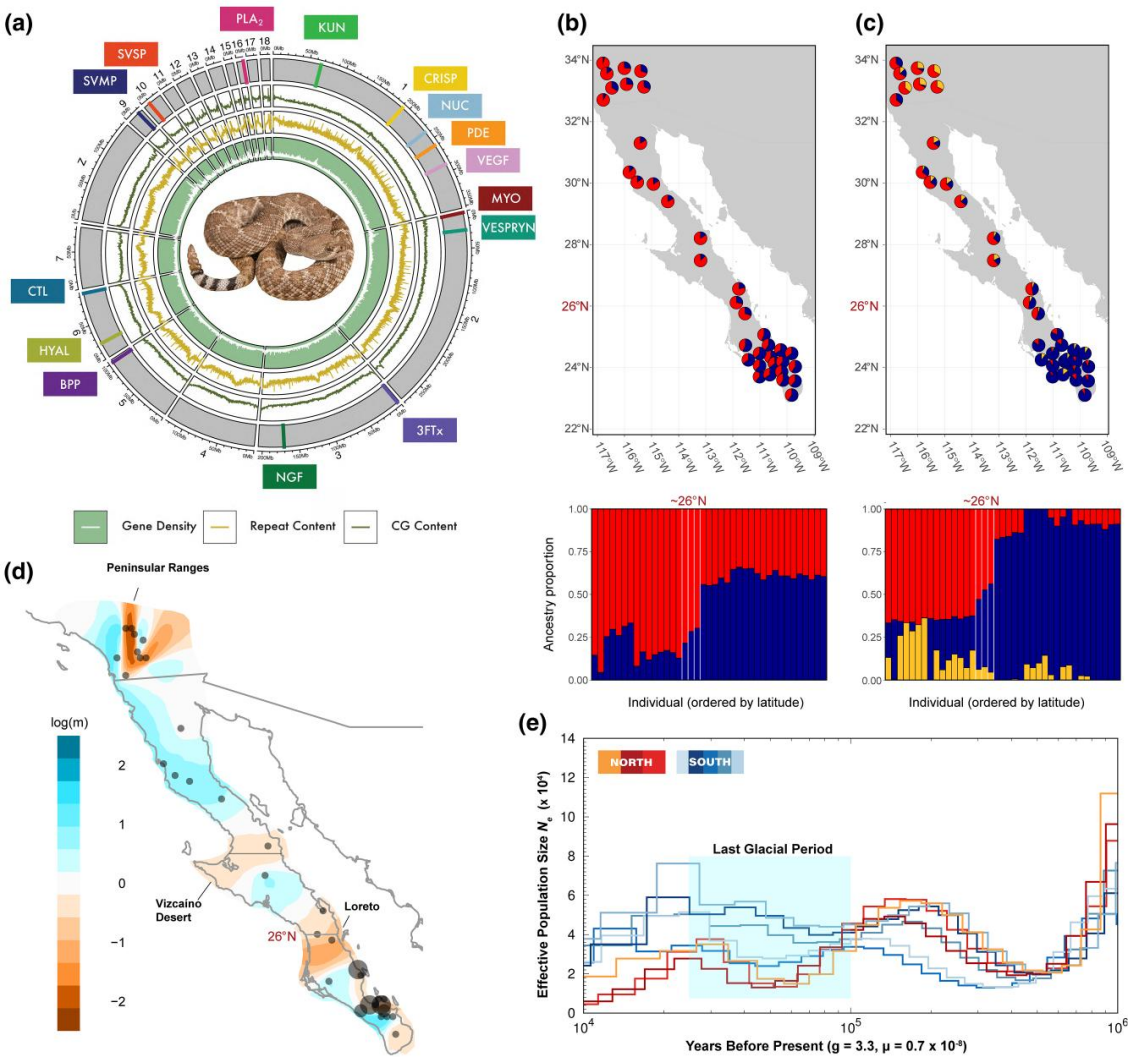
Reference genome assembly and genomic sequencing of *C. ruber* reveals two genetically distinct populations with unique demographic histories. a) Circos plot of the RagTag reference genome assembly displaying gene density, repeat content, CG content, and toxin gene families mapped to chromosome scaffolds as represented by corresponding colored lines. Toxin families are (ordered by chromosome): KUN, Kunitz-type toxin; CRISP, cytesine-rich secretory protein; NUC, nucleotidase; PDE, phosphodiesterase; VEGF, vascular endothelial growth factor; MYO, myotoxin; 3FTx, three-finger toxin; NGF, nerve growth factor; BPP, bradykinin-potentiating peptide; HYAL, hyaluronidase; CTL, C-type lectin; SVMP, snake venom metalloproteinase; SVSP, snake venom serine proteinase; ${\text{PLA}}_{2}$, phospholipase ${\text{A}}_{2}$. b–c) Population structure characterized from short-read WGS and ddRADseq data using ConStruct spatial models with b) K=2 and c) K=3. Maps depict individuals as pie charts reflecting ancestry proportions contributed by each genetic cluster. d) Estimated effective migration surface from WGS and ddRADseq data using EEMS. Shading indicates areas with relatively high (orange) and low (blue) landscape resistance to gene flow compared to a null area-wide model of isolation-by-distance (IBD). Plotted values of log(*m*) are effective migration rates relative to the overall migration rate across the study area. Circles represent sampling locations, and circle size corresponds to sampling density. e) Estimates of demographic histories across the two distinct populations from panel (b). Lines represent effective population size ($N_{e}$) estimated from eight individuals using a generation length of 3.3 years and a mutation rate of 0.007 per lineage per million years. Colors indicate $N_{e}$ estimates of individuals sampled from the northern population (warm) and southern population (cool; as determined in panel (b)). Contact zone (∼26^∘^CN) is indicated throughout.

We generated a reference *C. ruber* genome using PacBio HiFi reads (∼20× coverage) for a subadult male collected within the *C. r. ruber* range near Bahía de los Ángeles, Baja California, MX (Fig. 1). Genome assembly length was 1.59 Gb (1,126 contigs, N50 of 6.25 Mb, L50 of 65; Table 1). We calculated additional genome quality assessment metrics, such as phred quality score (55), k-mer completeness (96%), and BUSCO (96.5% complete Vertebrata; 93.0% complete Sauropsida; Table 1). To achieve a chromosome-level assembly, we scaffolded the *C. ruber* assembly to the chromosome-level assembly of the Eastern Diamondback Rattlesnake (*C. adamanteus*; Hogan et al. 2024) using RagTag (Alonge et al. 2022). The number of contigs in the assembly was reduced ∼10× to 111 scaffolds (N50 of 206.58 Mb), and all 17 autosomes assembled for *C. adamanteus* were assembled for *C. ruber*. Because our genome individual was male, only the Z sex chromosome was assembled (Fig. 2a). We annotated the genome and identified 20,771 protein-coding genes including 94 putative toxin genes within 14 toxin families (Fig. 2a). Multiple toxin families were found on microchromosomes (chromosomes 9–18 in Fig. 2a) as large tandem arrays, consistent with toxin genomic organization in other rattlesnakes (Schield et al. 2019; Margres et al. 2021a; Hogan et al. 2024).

**Table 1:** Genome assembly statistics for *C. ruber*

Metric	
Assembly size (Gb)	1.59
Number of contigs	1,126
Contig N50 (Mb)	6.25
Contig L50	65
Number of scaffolds	111
Scaffold N50 (Mb)	206.58
bp anchored to chromosomes (Gb)	1.57 (98.7%)
Phred quality score (Q)	55
k-mer completeness %	96
BUSCO Vertebrata (C — D — F — M) %	96.5 — 1.0 — 1.1 — 2.4
BUSCO Sauropsida (C — D — F — M) %	93.0 — 1.2 — 1.2 — 5.8
CG content, %	39.8
Repeat content, %	49.07
Protein-coding genes	20,771
Putative venom protein-coding genes	94

All metrics are for the *de novo* assembly except “Number of scaffolds”, “Scaffold N50”, and “bp anchored to chromosomes” which represent metrics for the RagTag assembly to *C. adamanteus*. BUSCO metrics are shown as complete (C), duplicated (D), fragmented (F), and missing (M). Genome assembly available at NCBI PRJNA1051499.

### Population Genomics Identifies Distinct Populations and Evolutionary Histories

We used conStruct (Bradburd et al. 2018) across 39 individuals (2,241 SNPs) to characterize population structure (Fig. 2b–c). Spatial models invariably had higher predictive accuracy than nonspatial models, with predictive accuracy reaching an asymptote at K=2-3 genetic clusters (supplementary fig. S1, Supplementary Material online). For the spatial models, additional genetic clusters beyond K=2 explained <5% of total genetic covariance, suggesting that K=2 was an appropriate choice for characterizing population genetic structure (Fig. 2b). After cross-validation, we fit final spatial models using the full dataset for K=2 and K=3. For K=2, populations were spatially sorted by latitude (Fig. 2b), with contact at ∼26^∘^CNN latitude, relatively consistent with current *C. ruber* subspecies delineation (Fig. 1; Grismer 2002). A similar pattern was observed for K=3 (Fig. 2c), with additional weak population structure at the northern range edge. We calculated the fixation index (${\text{F}}_{ST}$) between the populations for K=2 in conStruct (hereinafter referred to as the north and south populations) using the full genomic dataset (north n=19; south n=22; 5,284 SNPs) as well as the reduced genomic dataset (north n=18; south n=21; 2,241 SNPs) used specifically for conStruct. We found that ${\text{F}}_{ST}=0.295$ and 0.301, respectively. We also visualized patterns of sequence dissimilarity using the full genomic dataset (n=41; 5,284 SNPs) using PCoA. Individuals clustered according to the population structure identified in conStruct; southern individuals clustered tightly along both PCo1 and PCo2 while northern individuals clustered tightly along PCo1, but with increased variance along PCo2 (supplementary fig. S2A, Supplementary Material online).

Next, we estimated effective migration surfaces (EEMS; Petkova et al. 2016) using the full genomic dataset (n=41; 5,284 SNPs) to explore spatially variable migration rates across the landscape and visualize departures from IBD (Fig. 2d). We observed three areas of relative reductions in gene flow: (i) the Peninsular Ranges of Southern California, (ii) the Vizcaíno Desert of the Baja Peninsula, and (iii) the current *C. ruber* subspecies boundary at ∼26^∘^CNN latitude near the town of Loreto, BCS, MX (Fig. 2d).

Lastly, we estimated demographic histories for the north (n=3) and south (n=5) populations using the Pairwise Sequentially Markovian Coalescent model (PSMC; Fig. 2e; Li and Durbin 2011) on our whole-genome data. Effective population size ($N_{e}$) decreased in both populations between ∼100 and 200 ka before present and continued to decrease during the last glacial period (Broecker and Hemming 2001) between ∼50 and 100 ka for the northern population while stabilizing in the southern population (Fig. 2e).

### Venom Expression Varies Extensively Across Life History and Less So Across Geographic Space

We conducted a PCA on the venom proteomic data for 20 individuals (supplementary table S1, Supplementary Material online) and found that PC1 (65%) was primarily associated with SVL, with individuals clustering into two groups separated at ∼65 cm SVL (supplementary fig. S3, Supplementary Material online). Indeed, a linear regression showed that venom PC1 was significantly correlated with SVL (P<0.001, adj-$R^{2}=0.82$; supplementary fig. S3B, Supplementary Material online). To test for venom protein expression differentiation across age class (≤ 65 cm juvenile) and population (northern and southern populations as defined in conStruct), we conducted a PERMANOVA. Only ontogeny was significant (P<0.001, $R^{2}=0.65$; adult n=14; juvenile n=6); neither population (P=0.194, $R^{2}=0.03$; north n=11; south n=9) nor the interaction between age and population (P=0.275, $R^{2}=0.02$) were significant. Overall, our proteomic analyses revealed that, at the trait level, venom expression was significantly different between age classes but not significantly different between populations.

**Fig. 3.**
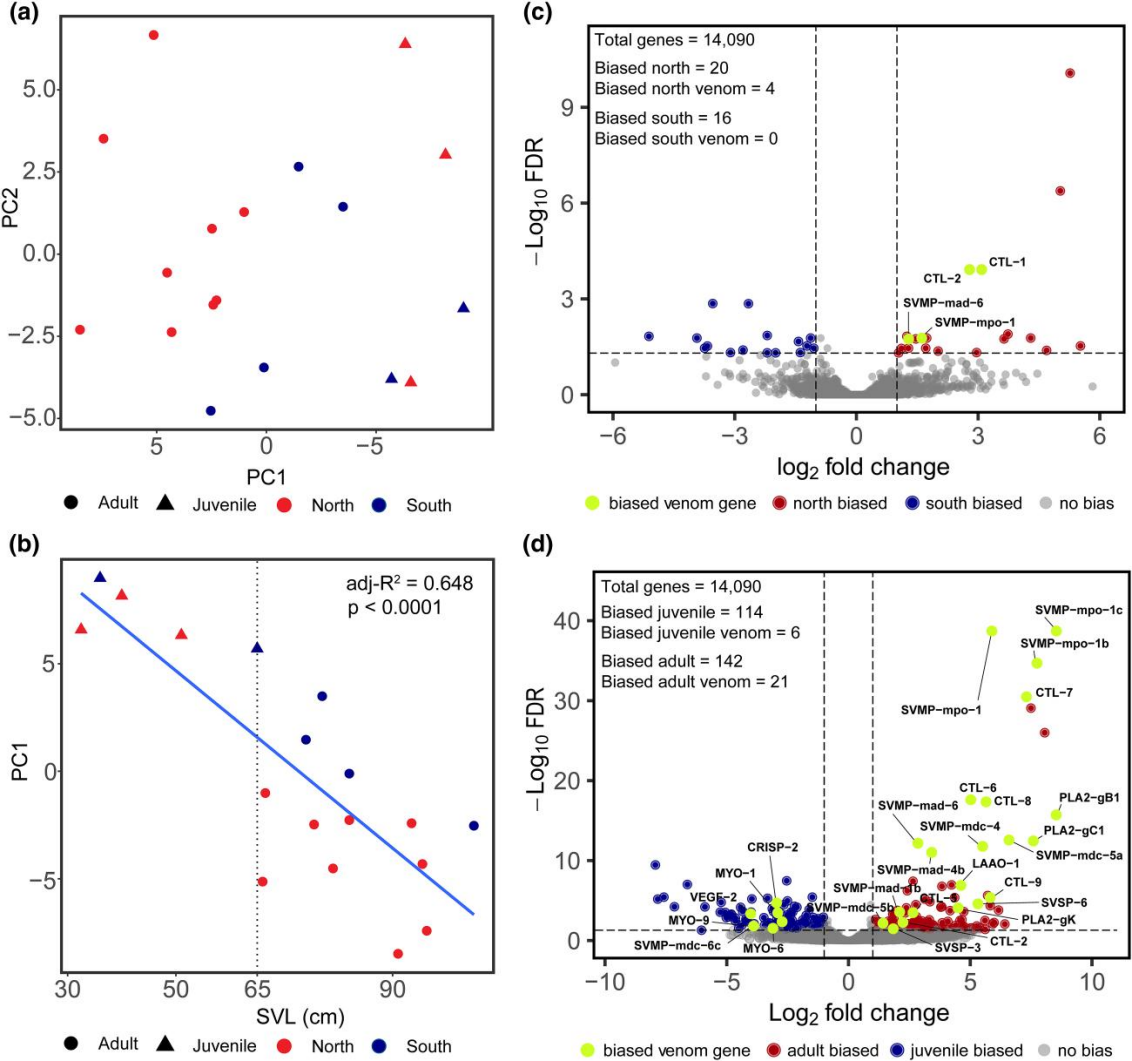
Differential venom expression across life history and geographic space in *C. ruber*. a) Principal Component Analysis of venom-gland transcriptome DESEQ2 normalized count data, and b) Regression of Principal Component 1 (PC1) with SVL. Dotted line at 65 cm SVL shows the cut-off used for age class designation. Proportion of variance accounted for in PC1 and PC2 was 31% and 13%, respectively. c–d) Volcano plots of differential expression calculated from DESeq2 between populations c) and age classes d). Vertical dotted lines represent LFC ≥ 1, and horizontal dotted line represents *α* ≤ 0.05. Green points in each plot denote significantly DE toxin transcripts, and their placement denotes group bias. SVL, snout–vent–length; BPP, bradykinin-potentiating peptide; CRISP, cytesine-rich secretory protein; CTL, C-type lectin; MYO, myotoxin; ${\text{PLA}}_{2}$, phospholipase ${\text{A}}_{2}$; SVMP, snake venom metalloproteinase; SVSP, snake venom serine proteinase.

To identify the specific toxin genes underlying ontogenetic venom variation and determine whether any individual toxin genes were significantly DE between populations, we generated venom-gland transcriptome data for 18 individuals across the range (Fig. 3). We first verified that the venom gland transcriptomic data exhibited similar patterns to those observed in the venom proteomic data by reconducting both PCA and PERMANOVA (Fig. 3a,b). PC1 (31%) was again significantly and positively correlated with SVL (P<0.001, adj-$R^{2}=0.65$; Fig. 3a), and only ontogeny was significant in the PERMANOVA (P=0.005, $R^{2}=0.31$; adult n=13; juvenile n=5); neither population (P=0.200, $R^{2}=0.07$; north n=12; south n=6) nor the interaction between age and population (P=0.590, $R^{2}=0.02$) were significant.

We identified specific genes that were significantly DE across populations (Fig. 3c) and age classes (Fig. 3d). Between populations (north n=12; south n=6), four toxin genes were significantly DE, with all four genes (*C-type lectin [CTL]–1, CTL–2, snake venom metalloproteinase [SVMP]–mad–6, SVMP–mpo–1*) exhibiting higher expression in the northern population. Between age classes (adult n=13; juvenile n=5), while accounting for population, 27 toxin genes were significantly DE. The majority (n=21) of the genes were biased toward adults (i.e. more highly expressed in adults than juveniles), with most genes belonging to the *SVMP* (n=9) and *CTL* (n=6) toxin families. Most juvenile-biased toxin genes (n=6) belonged to the myotoxin gene family (n=3). See supplementary table S2, Supplementary Material online for details of all DE transcripts between age groups and populations.

### Conditional Redundancy Analysis Identifies Life History as the Most Predominant Driver of Venom Evolution

To determine the relative roles of putatively neutral and adaptive evolutionary processes in driving venom expression evolution, we used conditional RDA to estimate the effects of nontoxin sequence variation (our proxy for neutrality; supplementary fig. S2A-C, S4, Supplementary Material online), toxin sequence variation, abiotic environmental factors, and prey data (availability and phylogenetic distance) on multivariate venom expression data.

First, we used PCoA to determine whether (i) nontoxin SNPs accurately reflected patterns of neutral genomic sequence variation and (ii) patterns of nontoxin sequence variation were robust to the inclusion of nonsynonymous variants. Patterns of sequence variation under PCoA were consistent among neutral genomic SNPs, nontoxin synoynmous SNPs, and nontoxin SNPs including all variant types (supplementary fig. S2, Supplementary Material online). Additionally, correlation between PCo1 of nontoxin synonymous SNPs and PCo1 of all nontoxin SNPs was highly significant (supplementary fig. S4, Supplementary Material online; P<0.001; $R^{2}=0.97$). Therefore, nontoxin SNPs including all variant types served as a valid proxy for neutral patterns of genetic divergence.

Using conditional RDA with toxin gene read count estimations from HTSEQ-count as the multivariate response variable, the full model, including all variables, was significant (P=0.002; adj-$R^{2}=0.73$; Table 2), indicating that our model captured at least one or more variables that significantly explained venom expression variation. The marginal (i.e. best) model (adj-$R^{2}=0.54$) as determined from forward model selection revealed that SVL (P=0.003; adj-$R^{2}=0.30$), prey availability (NMDS2; P=0.010; adj-$R^{2}=0.14$), and abiotic factors (Bioclim PC1; P=0.012; adj-$R^{2}=0.10$) were the most significant predictors of venom expression variation (Table 2).

**Table 2:** Results of the conditional RDA for venom-gland transcriptome normalized read count data from HTSeq-count as the response variable

	F	*P*-value	adj-$R^{2}$
Full Model	5.521	0.002	0.73
Marginal Model			0.54
SVL	8.32	0.003	0.30
Prey NMDS2	5.17	0.01	0.14
Bioclim PC1	4.49	0.012	0.10

Marginal model was identified using forward model selection on all explanatory variables. Results for all variables can be found in supplementary table S3, Supplementary Material online.

Similarly, using read count estimations from Stringtie2 as the multivariate response variable, the full model, including all variables, was again significant (P=0.003; adj-$R^{2}=0.66$; Table 3). The marginal model (adj-$R^{2}=0.62$) as determined from forward model selection differed slightly from the best model using HTSeq-count data as input; here, SVL (P=0.001; adj-$R^{2}=0.44$), abiotic factors (Bioclim PC1; P=0.001; adj-$R^{2}=0.12$), and nontoxin sequence variation (Nontoxin PCo1; P=0.020; adj-$R^{2}=0.06$) were the most significant predictors of venom expression variation (Table 3).

**Table 3:** Results of the conditional RDA for venom-gland transcriptome normalized read count data from stringtie2 as the response variable.

	F	*P*-value	adj-$R^{2}$
Full Model	4.29	0.003	0.66
Marginal Model			0.62
SVL	14.24	0.001	0.44
Bioclim PC1	5.38	0.001	0.12
Nontoxin PCo1	3.42	0.02	0.06

Marginal model was identified using forward model selection on all explanatory variables. Results for all variables can be found in supplementary table S3, Supplementary Material online.

### Life History Best Explains Expression Evolution Across Individual Toxin Gene Families

We determined whether expression variation of the six most abundantly expressed toxin families (bradykinin-potentiating peptide [BPP], C-type lectin [CTL], Myotoxin, phospholipase ${\text{A}}_{2}$ [${\text{PLA}}_{2}$], snake venom metalloproteinase [SVMP], snake venom serine proteinase [SVSP]) were significantly correlated with different explanatory variables. Variation across all toxin families, as identified in the marginal models, was significantly correlated with SVL (Table 4). Nontoxin sequence variation was also found to be a significant predictor of CTL, Myotoxin, and SVMP expression. Abiotic variation (Bioclim PC1) was the most significant predictor of ${\text{PLA}}_{2}$ expression variation. Prey was identified as a significant predictor of expression variation in BPP and SVSP toxin families, with prey availability (NMDS2) predicting BPP expression variation, and prey mean phylogenetic distance (MPD) predicting SVSP expression variation. See supplementary table S3, Supplementary Material online for detailed results of conditional RDAs for individual toxin families.

**Table 4:** Significant variables of the marginal models identified through forward model selection from conditional RDAs using the top six most abundantly expressed toxin families

Toxin Family	Marginal Model	*P*-value	adj-$R^{2}$
BPP	SVL	0.010	0.31
	Prey NMDS2	0.008	0.22
CTL	SVL	0.004	0.32
	Nontoxin PCo1	0.015	0.15
Myotoxin	SVL	0.004	0.34
	Nontoxin PCo1	0.039	0.13
${\text{PLA}}_{2}$	Bioclim PC1	0.001	0.38
	SVL	0.001	0.29
SVMP	SVL	0.007	0.27
	Nontoxin PCo1	0.013	0.18
SVSP	SVL	0.012	0.19
	Prey MPD	0.047	0.11

Results for all variables in each family can be found in supplementary table S3, Supplementary Material online.

## Discussion

### Assembly and Annotation of Reference Quality *C. ruber* Genome

Genomic content of the reference genome assembly was similar to that of other snake assemblies (Vonk et al. 2013; Yin et al. 2016; Schield et al. 2019; Suryamohan et al. 2020; Li et al. 2021; Margres et al. 2021a; Westeen et al. 2023; Hogan et al. 2024). Notably, the *C. ruber* genome assembly displayed improved contiguity compared to several prior *Crotalus* assemblies, exhibiting a higher contig N50 and fewer total contigs compared to *C. tigris* (Margres et al. 2021a) and *C. viridis* (Schield et al. 2019). Overall, the accurate and contiguous reference-quality genome for *C. ruber* enabled us to robustly explore the effects of multiple evolutionary processes on venom evolution using reference-based genomic and transcriptomic analyses.

### Population Genomics Reveals Two Genetically Distinct Populations with Unique Evolutionary Histories

We identified two genetically distinct populations separated by latitude with contact at ∼26^∘^CNN latitude near Loreto, BCS, MX (Fig. 2b), consistent with previous results (Harrington et al. 2018). Genetic differentiation between the two identified populations was extensive (${\text{F}}_{ST}=0.295\text{--}0.301$), with levels of fixation similar to that of highly genetically distinct populations of other North American vipers (Gibbs et al. 1997; Margres et al. 2019; Schmidt 2019). Reduced gene flow compared to expectations under a model of IBD was observed at the northeastern range edge near the Peninsular Ranges (Fig. 2d), which separate the California chaparral from the Sonoran Desert. The Sonoran Desert serves as a barrier to migration for many terrestrial organisms (Ernest et al. 2003; Brown et al. 2009), and for *C. ruber* (Greenberg 2002), the barrier likely exists due to climatic differences and competition with congenerics such as its sister taxon, the Western Diamondback Rattlesnake (*C. atrox*; Alencar et al. 2016). Reduced gene flow was also observed near the Vizcaíno desert (Fig. 2d). Numerous species of the Baja region exhibit population differentiation occurring at the Vizcaíno desert (Riddle et al. 2000). Three hypotheses suggest that this region may serve as a major barrier to migration in multiple organisms due to (i) a proposed ancient transpeninsular seaway that bisected the peninsula during the late Miocene to middle Pleistocene, (ii) isolation due to Pleistocene glacial–interglacial cycles, or (iii) differences in rainfall patterns between the peninsular regions (reviewed in Dolby et al. 2022). The Vizcaíno desert region, however, functions only as a minor barrier to migration in *C. ruber*, at least relative to the Peninsular Ranges and subspecies boundary at ∼26^∘^CNN latitude (Fig. 2d). The deviation of *C. ruber* population structure from the patterns exhibited by other species (Riddle et al. 2000) was not associated with any apparent current or ancient topographic or geographic barriers to dispersal; rather, population structure has been proposed to be potentially linked with climatic fluctuations that occurred during the Pleistocene, resulting in temporary isolation of the two populations ∼450–510 ka before present until secondary contact ∼80 ka before present (Harrington et al. 2018). $N_{e}$ in the northern and southern populations appeared to concordantly increase during the potential period of climate-driven isolation (∼200–450 ka before present). At the time of purported secondary contact during the last glacial period (∼80 ka before present), $N_{e}$ decreased in the northern population while remaining relatively stable in the southern population (Fig. 2e). The observed differences in $N_{e}$ between the two populations during the last glacial period suggests a pivotal role of climate-induced pressures on $N_{e}$ and migration dynamics. Climate conditions were likely less favorable for snake survival in the northern range during glacial periods (Herbert et al. 2001), potentially driving the previously isolated northern population south and leading to decreased $N_{e}$ and renewed contact with the southern population. Due to the limitations of PSMC in resolving more recent demographic histories, however, inferences of $N_{e}$ near the present may not be inferred accurately (Liu and Fu 2015; Nadachowska-Brzyska et al. 2016; Patton et al. 2019). Additional biogeographic analyses and sampling would be needed to further explore the distinct evolutionary histories of the two populations identified here.

### Venom Expression Differentiation Explained More by Ontogeny Than Genetic Population

Ontogenetic venom variation was much more pronounced than venom differentiation across populations. Indeed, age class explained ∼22× more variance in venom proteomic composition and ∼4× more variance in venom-gland transcriptome expression than population structure. The ontogenetic shift in venom expression occurred at ∼65 cm SVL (supplementary fig. S3, Supplementary Material online) with continued variance throughout the life history of an individual, similar to other *Crotalus* species (Schonour et al. 2020). Differential expression of individual genes revealed patterns of increased expression in SVMP and CTL toxin families in adults and the northern population and increased expression of myotoxins in juveniles. Myotoxins are small, basic peptides that induce physiologic tetanus of skeletal muscles, particularly in mice, and likely play an important role in subduing prey (Brenes et al. 1987; Mackessy et al. 2003; Mackessy 2021). SVMPs are a diverse family of large catabolic enzymes capable of causing severe damage to common structural proteins, inducing hemorrhage, and may aid in prey digestion (Kini and Koh 2016; Slagboom et al. 2017; Mackessy 2021). Variable ontogenetic and geographic expression of SVMPs and myotoxins is observed in multiple *Crotalus* species (Straight et al. 1991; Margres et al. 2015b; Smith et al. 2023), and such variation may be due to adaptive evolution. Adaptive differences may be produced by changes in prey preference at different life-history stages (Mushinsky et al. 1982) or optimal foraging strategy that promotes faster growth rates and reduces time spent in more vulnerable size classes (Werner and Gilliam 1984; Klauber 1997). For example, the production of large toxin enzymes such as SVMPs may be more metabolically costly (Mackessy 1988), leading to limited expression in juveniles. Although the precise mechanism remains unknown, the venom phenotype was significantly variable across age classes with only a limited number of toxins exhibiting differential expression across populations, suggesting that changes in venom expression due to maturity may have greater ecological implications (i.e. differences in prey size and/or species) compared to changes across populations.

### Venom Variation Across Space Explained Primarily by Ontogeny with Significant but Reduced Effects of Other Selective Pressures and Neutral Processes

#### Venom Variation Best Explained by Snake Size

Conditional redundancy analysis integrating snake size, environmental factors, prey availability, and prey phylogenetic distance revealed that snake size (i.e. ontogeny) best predicted multivariate venom expression variation, regardless of which read count estimation method was employed, consistent with our venom analyses described above. Similar to geographic venom variation, ontogenetic venom variation is commonly attributed to selection (Andrade and Abe 1999; Gibbs et al. 2011; Webber et al. 2016; Cipriani et al. 2017). Snakes, as gape limited predators, may select prey at different life-history stages (Shine 1991); therefore, the venom phenotype may adaptively shift as size increases to more effectively subdue and/or digest different, larger prey species (Margres et al. 2015b). Variable efficacy of adult and juvenile venom in differing prey items is observed in multiple snake species (Mackessy 1988; Andrade and Abe 1999; Margres et al. 2016b; Cipriani et al. 2017; Borja et al. 2018), suggesting that ontogenetic venom variation is often adaptive; however, the potential for neutral ontogenetic variation in snake venom has yet to be explored. Ontogeny may simply reflect developmental constraints which prevent the expression of otherwise beneficial traits or genes due to undeveloped key features or pathways (Gould and Lewontin 1979; Fernandez-Lorenzo et al. 1999; Barton and Boege 2017). Indeed, similar to other rattlesnakes (Margres et al. 2015b; Schonour et al. 2020; Hogan et al. 2024), we found that juvenile *C. ruber* venoms were simpler than adult venoms, with many more toxins upregulated in adults relative to juveniles (Fig. 3e). Despite the current lack of understanding on developmental constraints in snake venom, a better comprehension of the regulatory architecture underlying ontogenetic venom variation (Hogan et al. 2024) will enable future venom studies to incorporate such constraints into analyses of venom ontogeny.

Environmental differences also significantly explained venom expression variation using both read count estimation methods, consistent with previous work in other venomous snake species (Strickland et al. 2018; Margres et al. 2021b; Siqueira-Silva et al. 2021). Overall, variation in annual temperature and temporal fluctuations in temperature were the most important environmental factors (supplementary table S4, Supplementary Material online; PC1). Snakes further north experience cooler overall temperatures and greater annual temperature fluctuations compared to snakes in the south which experience consistently warmer temperatures throughout the year. Climactic factors such as temperature have been found to influence snake feeding behavior and prey preferences (Vincent and Mori 2008) which may in turn favor increased or decreased expression of certain toxin families that lead to more efficient feeding in particular climates. As described above, large toxin enzymes may aid in digestion; therefore, increased expression of these enzymes may be beneficial for snakes attempting to consume prey in cooler climates. Large enzymes such as SVMPs were more highly expressed in venoms from the northern population (Fig. 3c), suggesting a potential correlation between expression of putatively digestion-aiding toxin enzymes and cooler temperatures. Alternatively, environmental abiotic factors may have more accurately captured changes in prey availability across geographic space (see below), suggesting that venom expression variation corresponded with environmentally induced changes in prey availability. More detailed dietary analysis and toxicity measurements of different venoms in different prey under varying environmental conditions (e.g. assays conducted under different temperatures) would be needed to disentangle biotic and abiotic contributions to venom evolution.

Differences in prey availability were identified as significant within the marginal model using HTSeq-count derived data. Here, the significance of prey was primarily associated with an increase in prey availability at the northern range edge compared to individuals found throughout the Baja California Peninsula (supplementary table S5, Supplementary Material online; NMDS2). Venom composition and variation is frequently associated with differences in prey availability among populations (Daltry et al. 1996; Barlow et al. 2009; Gibbs and Mackessy 2009; Holding et al. 2016; Margres et al. 2017a; Smiley-Walters et al. 2017; Robinson et al. 2021; Smith et al. 2023), and variation in the number of available prey species between *C. ruber* populations appeared to contribute, in part, to venom evolution. Variables of prey availability and prey mean phylogenetic distance (MPD) within our model, however, assumed (i) that all *C. ruber* would consume a given prey item if present within its geographic location, and (ii) all prey are equally abundant at each location. We acknowledge that these assumptions ignore ontogenetic changes in prey preference and/or geographic variation in prey abundance (Andrade and Abe 1999; Mackessy et al. 2006; Dugan and Hayes 2012; Cipriani et al. 2017). Additional diet information, including precise characterization of changes in prey composition across life-history stages and variation in abundance for each prey species across space, would be necessary to confirm size/geographic-induced dietary constraints or preferences here.

Lastly, nontoxin sequence variation was identified as a significant predictor of multivariate venom expression variation with read count estimation from stringtie2. Although it was the weakest predictor of venom expression variation (adj-$R^{2}=0.06$) compared to ontogeny, abiotic factors, and biotic factors, its presence in the marginal model suggested that neutral evolutionary processes minimally explain some variation in the overall venom phenotype. Therefore, neutral evolutionary processes may have a diminished yet still significant impact on venom evolution. Significance of nontoxin sequence variation within the model, however, may be potentially confounded by strong population structure (Fig. 2; Holding et al. 2018); such population structure may have been the product of geographically limited dispersal and genetic drift, and/or may be due to selective pressures causing reduced immigrant fitness (Garant et al. 2007). Still, results of the marginal model suggested that neutral sequence variation, our proxy for neutral evolutionary processes, significantly explained some variation in the overall venom phenotype.

Although both read-count methods identified SVL as the most significant predictor of venom expression variation, the other significant predictors and their contributions to the model varied between the two methods. Specifically, nontoxin sequence variation was only a significant predictor for all toxins when using StringTie2 estimates; however, it was also a significant predictor across three specific toxin families (CTL, SVMP, and myotoxin) when using HTSeq-counts (Table 4). The significance of nontoxin sequence variation across both read-count methods provided confidence that the result was robust to any potential biases across methods. Why such differences occurred is not immediately clear, but varying sensitivities of the methods to different aspects of the data or inherent differences in how these methods process read counts were suspected (See Materials and Methods). Further evaluation of each method, potentially including additional datasets and validation of findings through complementary approaches, would be necessary to better understand these discrepancies.

#### Life History and Differing Secondary Factors Independently Contribute to Individual Toxin Family Evolution

Individual components of a complex trait like venom, such as specific toxin gene families, may evolve independently (Casewell et al. 2011, 2020; Schield et al. 2022); certain toxin families may play a more important role in specific aspects of feeding such as subduing, tracking, or digesting prey (Mackessy 2021), leading to unique evolutionary trajectories from different evolutionary mechanisms. For example, prey resistance to certain toxins or toxin families (Holding et al. 2016; Margres et al. 2017a; Gibbs et al. 2020; Robinson et al. 2021) may lead to variable expression of those toxins, whereas other toxins may evolve in response to abiotic conditions such as temperature (Tsai et al. 2003; Strickland et al. 2018; Margres et al. 2021b).

We tested whether variation across individual toxin families was best explained by distinct factors compared to multivariate venom expression variation. SVL was identified in the marginal models of all toxin families individually, further demonstrating the significance of ontogenetically induced venom variation in *C. ruber*. Variation in three of the toxin families (SVMP, CTL, myotoxin) was also significantly correlated with nontoxin sequence variation in addition to SVL (Table 4), suggesting that neutral evolutionary processes may contribute to variation across highly expressed toxin families of the venom phenotype. The relationship, however, may have been confounded by strong population structure (see above). Variation in the ${\text{PLA}}_{2}$ family was more significantly associated with environmental factors, particularly temperature, than SVL. Correlation between ${\text{PLA}}_{2}$ expression and environmental factors, especially those related to temperature, has been found in other Viperidae species (Tsai et al. 2003; Strickland et al. 2018; Margres et al. 2021b) and may be associated with temperature-driven variation in snake feeding behavior, prey availability, and/or prey preference (Vincent and Mori 2008). Consistent correlation observed across multiple species strongly implies a link between ${\text{PLA}}_{2}$ expression and environmental factors. Prey availability and prey phylogenetic distance was identified as a significant predictor of expression variation across the BPP and SVSP toxin families, suggesting that the evolution of these families may be strongly linked with prey-induced selective pressures.

The inclusion of snake size in the marginal models for all of the most abundantly expressed toxin families was concordant with patterns of venom expression variation, highlighting the importance of life history in shaping venom evolution in *C. ruber*. However, variation of secondary factors identified in the marginal models across multiple toxin families, such as BPPs, SVSPs, and ${\text{PLA}}_{2}$s, prompts further investigation into (i) why certain toxin families exhibit distinct putative selection pressures, and (ii) whether these toxin families exhibit similar patterns across multiple species.

## Conclusion

We sequenced and assembled the genome of *C. ruber*, characterized range-wide genetic and venom differentiation, and robustly explored the underlying factors associated with venom expression evolution, including neutral evolutionary processes. Venom variation was most significantly and overwhelmingly predicted by snake size; variation across life history may be the result of selection due to differences in prey and/or optimal foraging strategies (Adriaens et al. 2001; Hintz and Lonzarich 2018) or neutral mechanisms such as developmental constraints (Fernandez-Lorenzo et al. 1999; Barton and Boege 2017). Additional information on changes in diet preference across life history, functional data of venom toxicity in these prey, and characterization of the regulatory architecture underlying venom expression differentiation across age classes (e.g. Hogan et al. 2024) is needed to further explore the ultimate and proximate mechanisms driving ontogenetic venom variation in *C. ruber*. Although we also found that venom variation was significantly associated with abiotic and biotic factors, neutral patterns explained some variation in the venom phenotype and minimally warrant consideration and inclusion in future models.

By incorporating proxies for neutral and adaptive processes into a singular statistical framework, our study robustly shows the pivotal role of adaptive evolution in snake venoms, consistent with decades of research (Daltry et al. 1996; Sasa 1999; Mackessy et al. 2003; Sanz et al. 2006; Barlow et al. 2009; Vonk et al. 2013; Holding et al. 2016, 2018, 2021; Cipriani et al. 2017; Margres et al. 2017a; Smiley-Walters et al. 2017; Strickland et al. 2018; Davies and Arbuckle 2019; Arbuckle 2020; Casewell et al. 2020; Schonour et al. 2020; Margres et al. 2021b; Siqueira-Silva et al. 2021; Mason et al. 2022; Rao et al. 2022; Schield et al. 2022; Smith et al. 2023). However, several of these previous studies did not adequately account for neutral processes, providing reduced confidence in adaptive interpretations. We acknowledge that our findings are based on the analysis of a single species and trait, and neutral processes may play a larger role in shaping phenotypic variation in other species and biological traits crucial to fitness and survival (Wright 1931; Nei 2005; Ho et al. 2017). Consequently, accounting for the influence of neutral evolutionary processes remains critical when investigating the forces producing trait variation, particularly within species. Our findings, together with those of others (e.g. Aird et al. 2017; Hague et al. 2020), underscore the necessity of considering the complexity of evolutionary processes when investigating phenotypic evolution.

## Supplementary Material

evae198_Supplementary_Data

## Data Availability

The data underlying this article are available in its online Supplementary material and the National Center for Biotechnology Information (NCBI). All sequencing data generated in the study were submitted to NCBI under BioProject (PRJNA1051499). Accession numbers can be found in supplementary table S6, Supplementary Material online. Metadata are provided in supplementary tables S1, S6, and S7, Supplementary Material online. Ecological data were obtained from publicly available databases and all analytical softwares are publicly available.
