# Supplementary material for: Where the “ruber” Meets the Road: Using the Genome of the Red Diamond Rattlesnake to Unravel the Evolutionary Processes Driving Venom Evolution
Source: Genome Biol Evol. 2024 Sep 10;16(9):evae198. doi: 10.1093/gbe/evae198 (PMC11440179; doi:10.1093/gbe/evae198)
Supplement: evae198_Supplementary_Data [file evae198_supplementary_data.zip › Cruber_Supplementary_Figures.pdf]

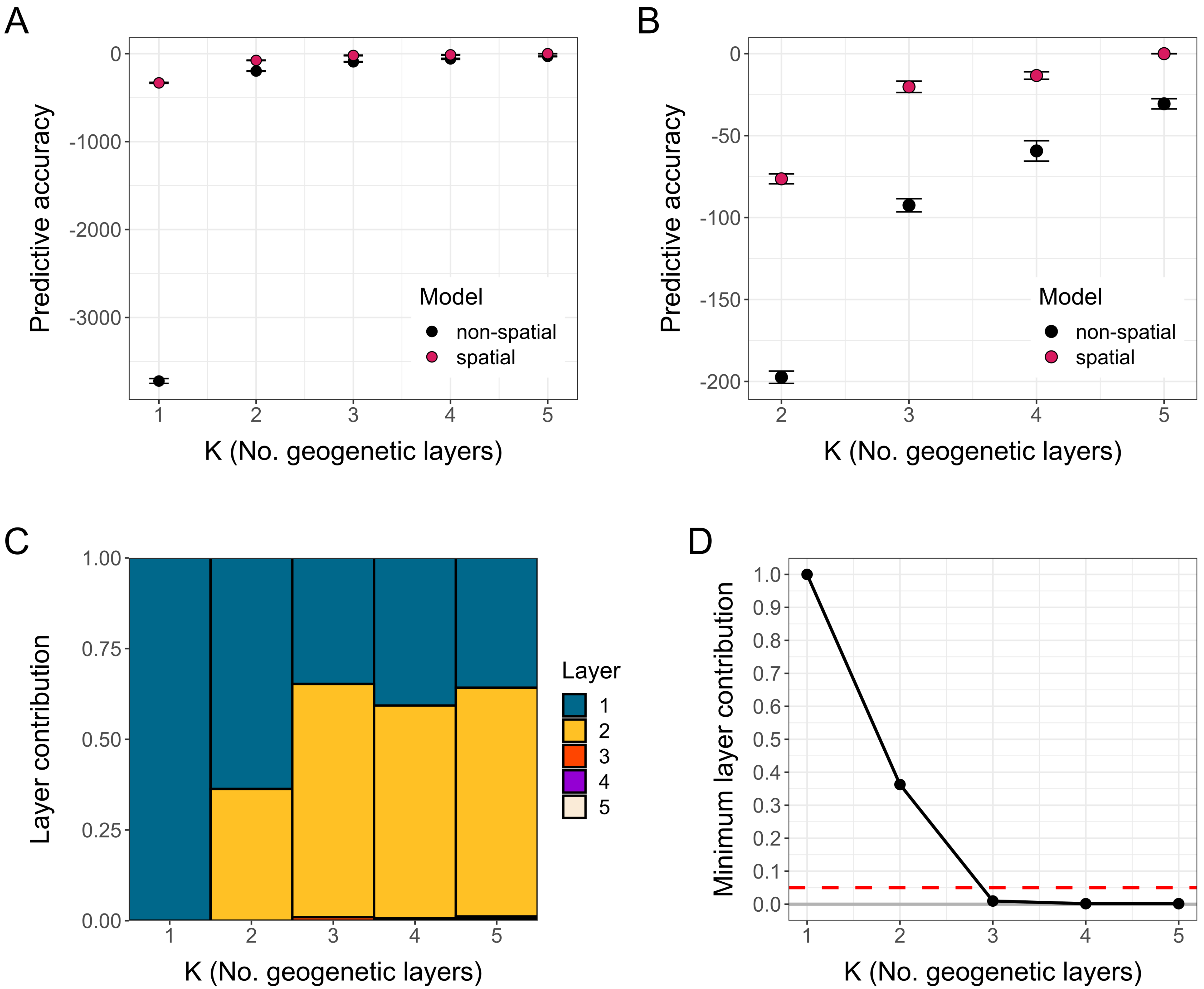

Fig. S1 - *ConStruct* model comparison using cross-validation. A) Predictive accuracy of spatial and non-spatial models for K = 1 – 5. Error bars show 95% confidence intervals based on 20 replicates. B) Contents of panel A for K = 2 – 5. C) Contributions of geogenetic layers (genetic clusters) to total genetic covariance for K = 1 – 5 using the spatial model. D) Smallest contribution of all layers for a given value of K. The red dashed line indicates a 5% contribution threshold.

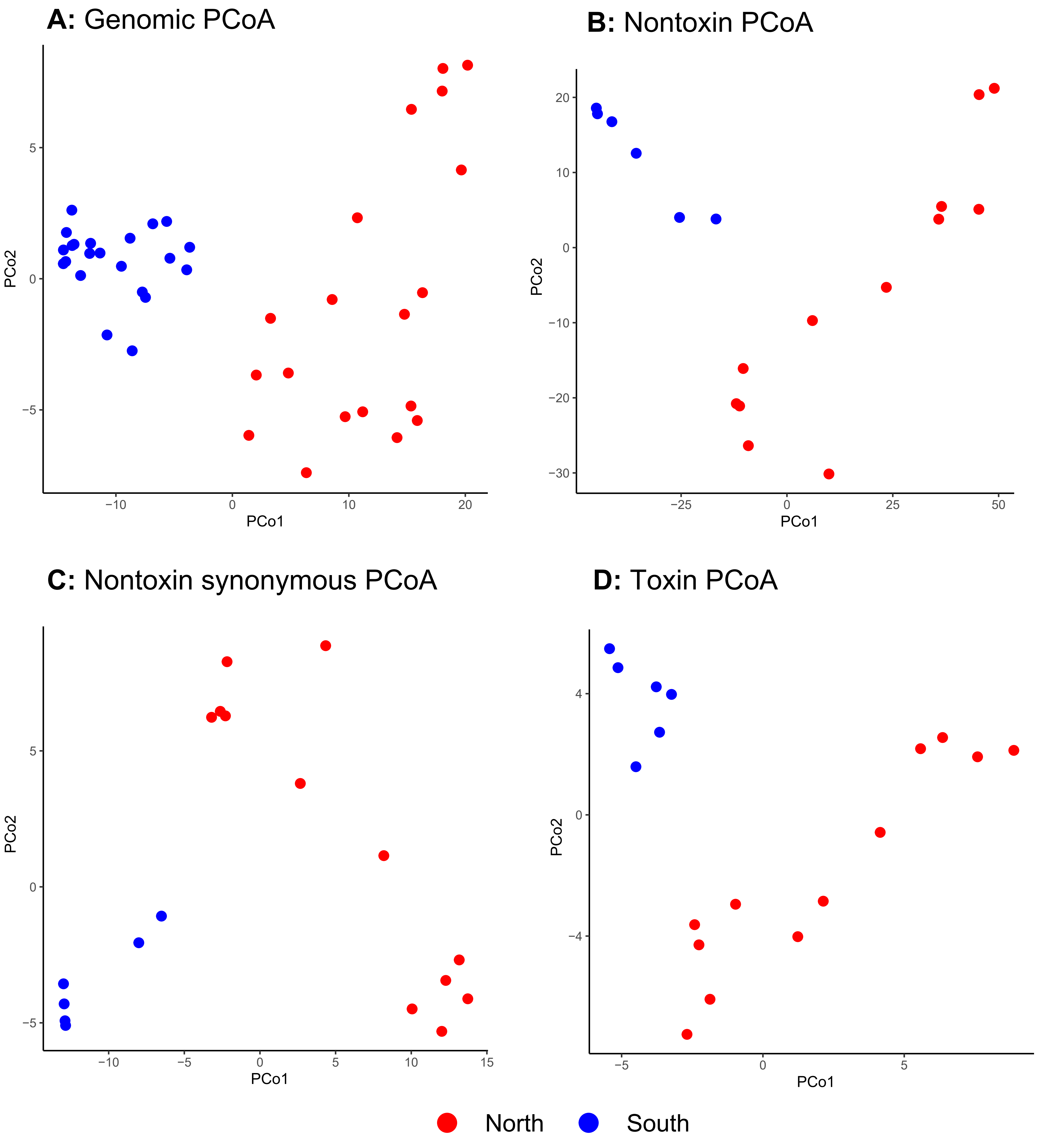

Fig. S2 – Principal coordinate analysis (PCoA) of (A) 5,284 SNPs across 41 individuals from combined ddRADseq and WGS data, (B) 41,236 SNPs from nontoxin venom gland transcriptomic data across 18 individuals, (C) 3,218 synonymous SNPs from nontoxin venom gland transcriptomic data across 18 individuals, and (D) 1,760 SNPs from toxin venom gland transcriptomic data across 18 individuals. Each point represents a single individual with colors denoting population. Eigenvalues for PCo1 and PCo2 were (A) 1,444 and 14 (B) 1,012 and 274, (C) 91 and 25, and (D) 20 and 15, respectively.

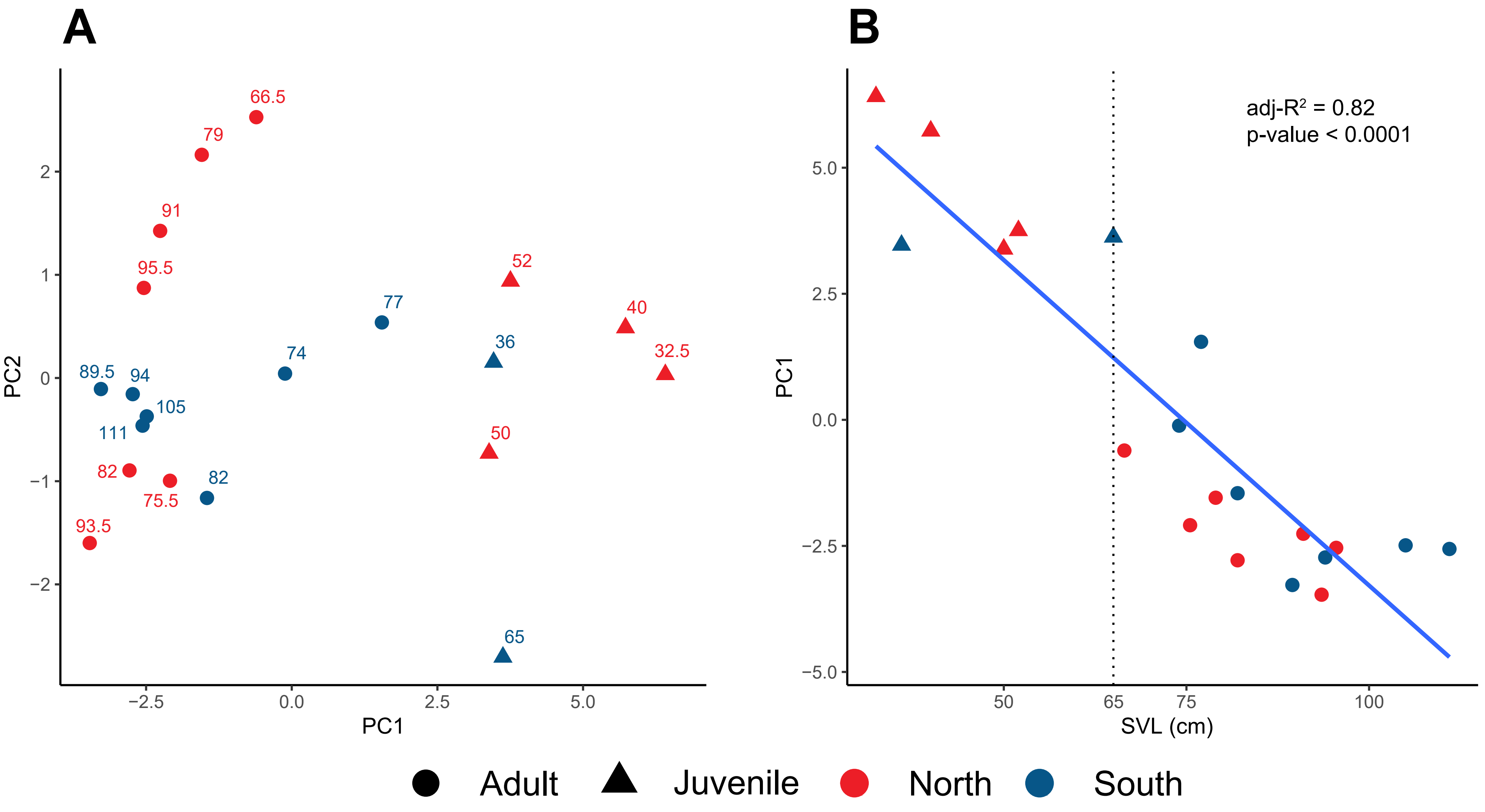

Fig. S3 – Principal component analysis (PCA) of venom Reversed-phase high-performance liquid chromatography (RP-HPLC) data. A) Plot of PC1 and PC2. B) Regression of PC1 with snake snout vent length (SVL). Each point represents a single individual with snake snout-vent length (SVL) indicated; triangles as juveniles (≤ 65 cm SVL), circles as adults (> 65 cm SVL), red as individuals from the northern population, and blue as individuals from the southern population. Proportion of variance explained by PC1 and PC2 was 0.65 and 0.10, respectively.

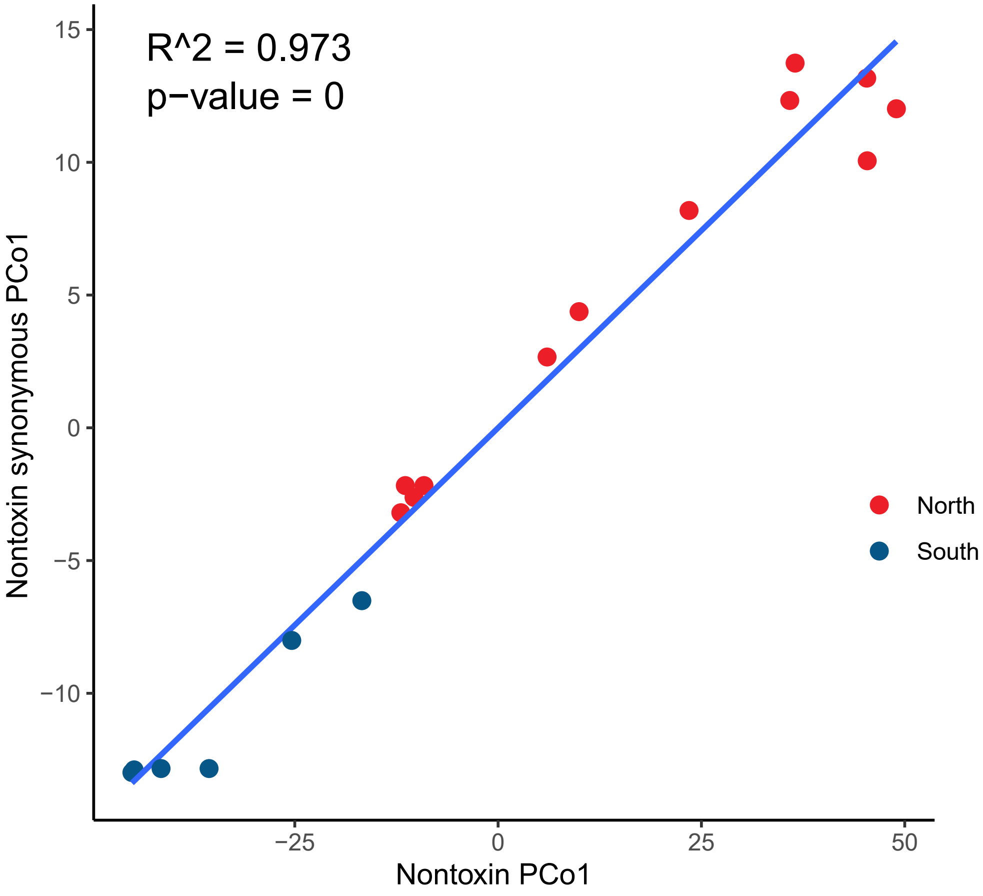

Fig S4 -- Regression of of PCo1s (Figure S3) from PCoA of 41,236 SNPs from nontoxin venom gland transcriptomic data (x-axis) and 3,218 synonymous only SNPs from nontoxin venom gland transcriptomic data (y-axis) across 18 individuals. Each point represents a single individual with colors denoting population

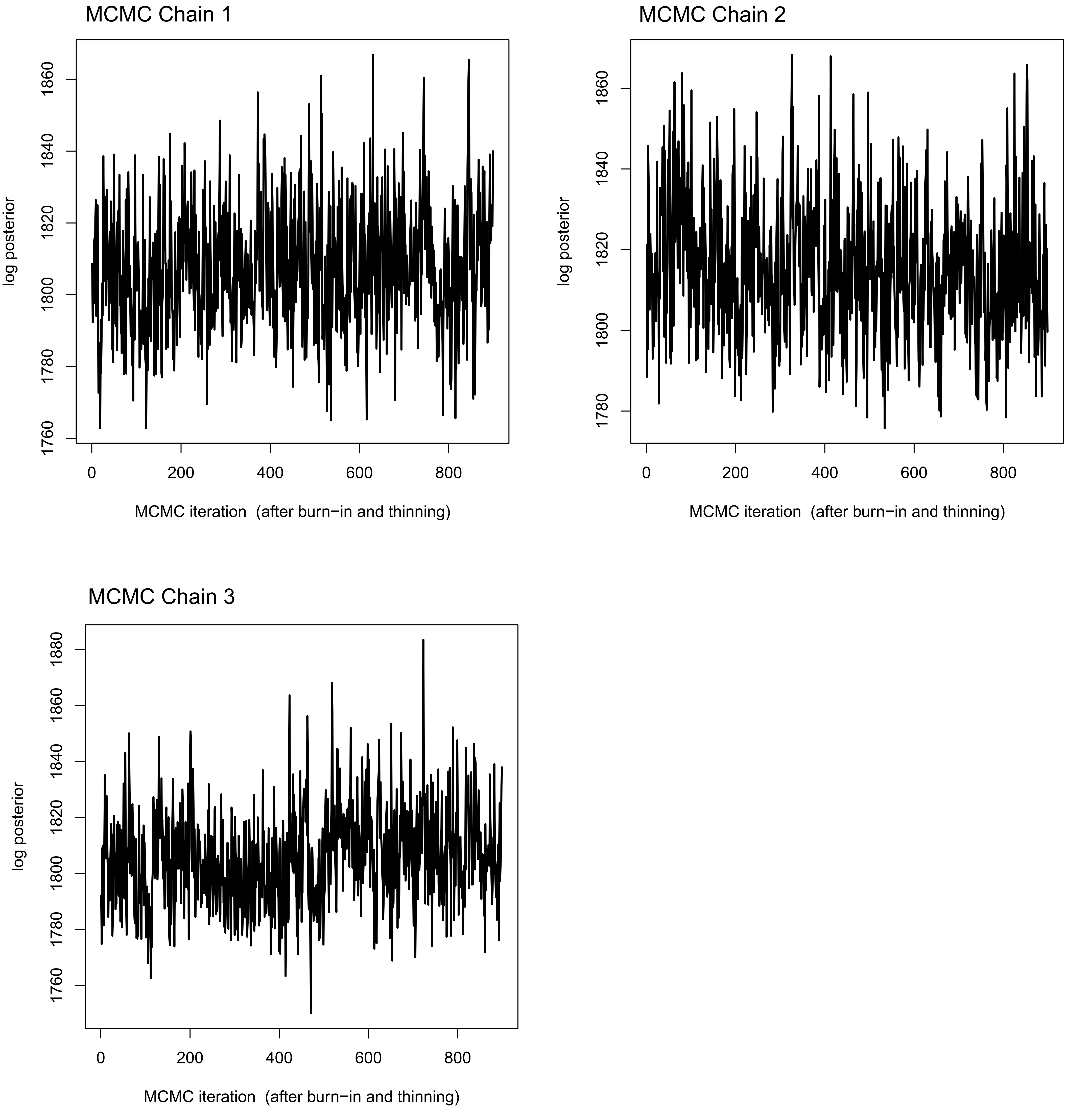

Fig. S5 – MCMC chains from EEMS used to estimate migration surfaces across three independent chains, each with 1,000 demes, 10,000,000 MCMC iterations, 1,000,000 iterations of burn-in, and a thinning interval of 10,000.

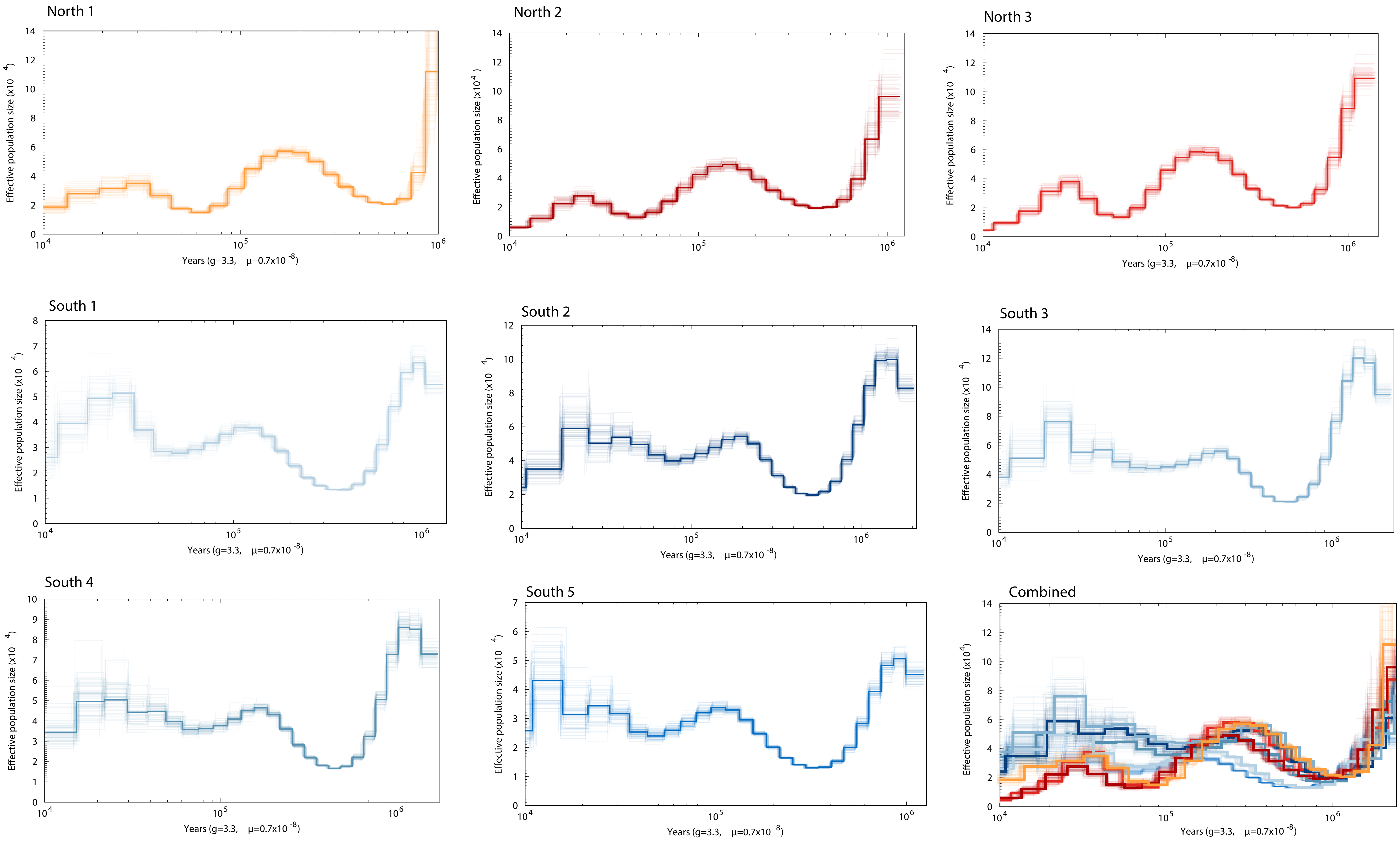

Fig. S6 – Pairwise sequencially Markovian coalescence (PSMC) for individual whole-genome samples. The data shown here were merged on the same graph for Figure 2E but are shown as individual graphs with bootstrapping of 100 replicates. The final panel shows all data, including 100 bootstrap replicates, merged on the same graph.

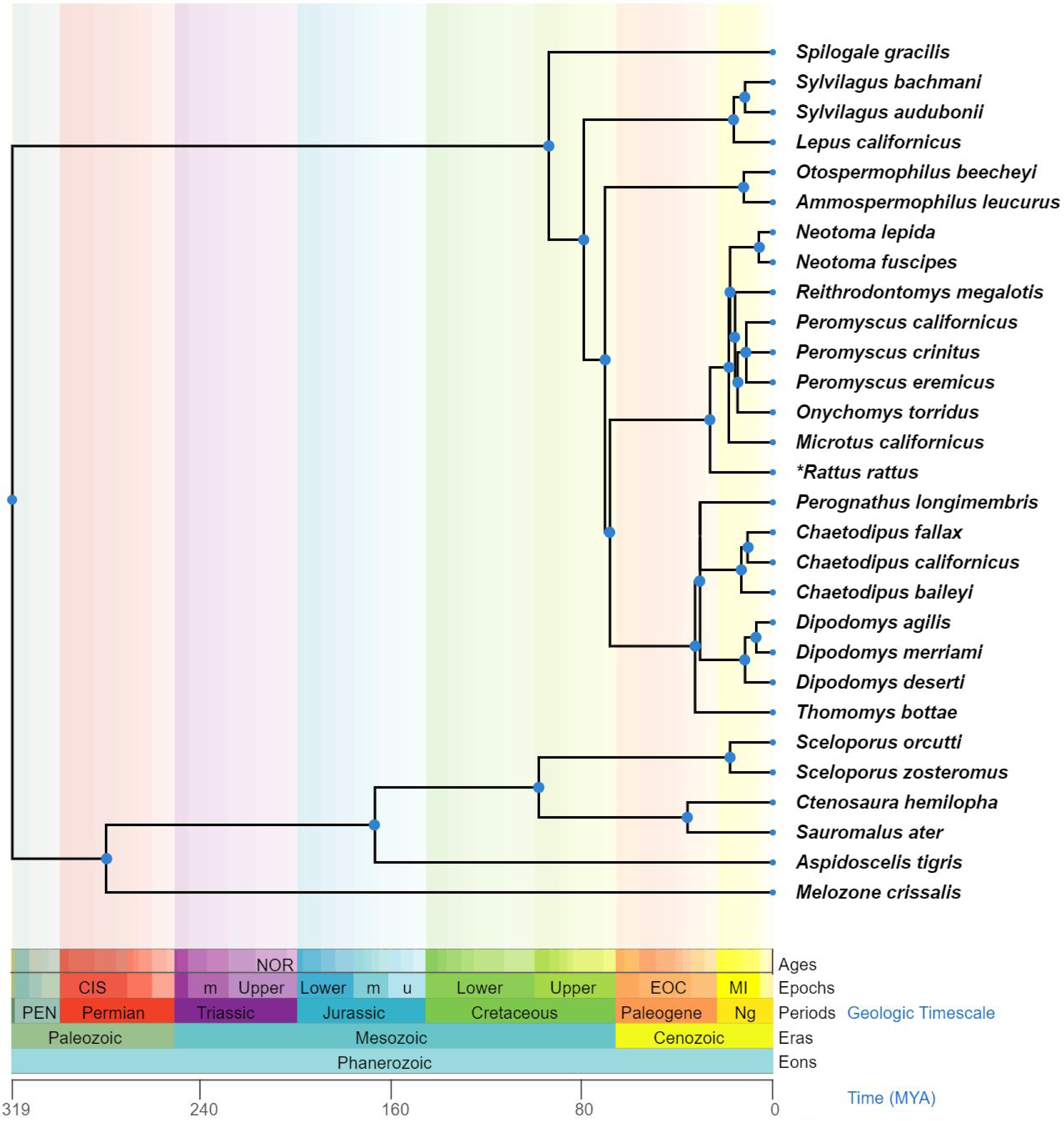

Fig. S7 –Phylogenetic tree generated of the 29 identified *C. ruber* prey items using www.timetree.org.
